# Bortezomib Augments Natural Killer Cell Targeting of Stem-Like Tumor Cells

**DOI:** 10.3390/cancers11010085

**Published:** 2019-01-14

**Authors:** Jesus I. Luna, Steven K. Grossenbacher, Ian R. Sturgill, Erik Ames, Sean J. Judge, Lyes A. Bouzid, Morgan A. Darrow, William J. Murphy, Robert J. Canter

**Affiliations:** 1 Department of Dermatology, University of California Davis Medical Center, Sacramento, CA 95817, USA; jiluna@ucdavis.edu (J.I.L.); skgrossenbacher@gmail.com (S.K.G.); irsturgill@ucdavis.edu (I.R.S.); erikames@gmail.com (E.A.); wmjmurphy@ucdavis.edu (W.J.M.); 2 Department of Surgery, Division of Surgical Oncology, University of California Davis Medical Center, Sacramento, CA 95817, USA; sjjudge@ucdavis.edu; 3 Department of Biological Sciences, California State University Sacramento, Sacramento, CA 95817, USA; lyesbouzid@gmail.com; 4 Department of Pathology and Laboratory Medicine, University of California Davis Medical Center, Sacramento, CA 95817, USA; madarrow@ucdavis.edu; 5 Department of Internal Medicine, University of California Davis Medical Center, Sacramento, CA 95817, USA

**Keywords:** natural killer cells, bortezomib, cancer stem cell, ALDH, sarcoma, brain tumor, pancreatic cancer

## Abstract

Tumor cells harboring stem-like/cancer stem cell (CSC) properties have been identified and isolated from numerous hematological and solid malignancies. These stem-like tumor cells can persist following conventional cytoreductive therapies, such as chemotherapy and radiotherapy, thereby repopulating the tumor and seeding relapse and/or metastasis. We have previously shown that natural killer (NK) cells preferentially target stem-like tumor cells via non- major histocompatibility complex (MHC) restricted mechanisms. Here, we demonstrated that the proteasome inhibitor, bortezomib, augments NK cell targeting of stem cell-like tumor cells against multiple solid human tumor-derived cancer lines and primary tissue samples. Mechanistically, this was mediated by the upregulation of cell surface NK ligands MHC class I chain-related protein A and B (MICA and MICB) on aldehyde dehydrogenases (ALDH)-positive CSCs. The increased expression of MICA and MICB on CSC targets thereby enhanced NK cell mediated killing in vitro and ex vivo from both human primary tumor and patient-derived xenograft samples. In vivo, the combination of bortezomib and allogeneic NK cell adoptive transfer in immunodeficient mice led to increased elimination of CSCs as well as tumor growth delay of orthotopic glioblastoma tumors. Taken together, our data support the combination bortezomib and NK transfer as a strategy for both CSC targeting and potentially improved outcomes in clinical cancer patients.

## 1. Introduction

Originally identified based on the ability of a small, phenotypically pure population of cancer cells to form heterogeneous tumors composed of diverse cell progeny, stem-like tumor cells have been identified in numerous solid and hematological cancer types [1]. The first published reports of tumor initiating cells were based on the isolation of CD34+ hematopoietic cells from blood samples of acute myeloid leukemia patients, which, once purified based on cell surface phenotype, were able to engraft into severely immunodeficient mice, differentiate into phenotypically distinct progeny, and demonstrate self-renewal capabilities [1,2]. Follow up studies have identified stem-like tumor cells in multiple mammalian tumor types, including human breast cancer, head and neck cancers, brain cancer, pancreatic cancer, melanoma, sarcoma, multiple myeloma, colon cancer, liver cancer, and kidney cancers, among others [3]. Although cancer stem cells (CSCs) have been challenging to phenotypically categorize, multiple studies have relied on the expression and/or co-expression of specific cell surface and intracellular proteins in a variety of tumor models. However, a major challenge in the study of CSCs is the lack of a reliable, uniform, and practical marker system which can be used to identify and phenotype CSCs across different tumor types [4]. The activity and expression of the family of intracellular enzymes known as aldehyde dehydrogenases (ALDH) is one of a select few cellular constituents that has been found to reproducibly correlate with a CSC phenotype across tissue types [5,6].

In fact, ALDH activity has been shown to identify stem-like tumor cells with CSC behavior in diverse solid tumor types [7,8,9,10,11,12,13]. In addition, the clinical relevance of ALDH expression has been evaluated in several scenarios, and ALDH expression has been found to correlate with worse overall survival for patients with non-small cell lung cancer, breast cancer, and ovarian cancer [14]. For instance, it has been described that *ALDH1A1* and *ALDH1B1*, two members of the ALDH gene superfamily composed of 17 functional genes and three pseudogenes, are associated with hematopoietic stem cells and cancer stem cell functions, respectively [15,16]. Taken together, these data reinforce the significance of therapeutically targeting ALDH positive cancer cells (which are often resistant to conventional cytotoxic cancer treatments) as well as tumors with high ALDH expression.

Recently, our lab has demonstrated that immunotherapy using isolated, expanded, and activated human natural killer (NK) cells is able to selectively target human CSCs, including those with an ALDH^bright^ phenotype [17]. While NK cells are known to possess the ‘innate’ ability to kill tumor cell targets in a major histocompatibility complex (MHC)-unrestricted fashion, it is the expression of stress ligands, such as MHC class I chain-related protein A and B (MICA and MICB), and apoptosis-inducing receptors, such as Death receptor (DR) 5, that appear to drive CSC susceptibility to NK cell targeting and killing. Overall, NK cell activity and cytotoxic function is tightly regulated by the balance of activating and inhibitory signals received upon engagement with a target cell [18,19], and CSCs have been found to express higher levels of these activating receptors compared to non-CSCs within the same tumor [17,20].

Bortezomib is a first-generation proteasome inhibitor used clinically for the treatment of patients with multiple myeloma and mantle cell lymphoma. One of the proposed mechanisms of action of the drug is the potentiation of NK cell killing towards target cells. Indeed, bortezomib has been demonstrated by our lab and others to promote NK cell lysis of target cells via the upregulation of death receptors on the tumor cell surface and through the promotion of caspase activation [21,22,23,24,25]. However, it is unclear how bortezomib may influence the susceptibility of ALDH^bright^ cells to NK cell killing. While we have previously shown that radiotherapy is capable of sensitizing these cells to NK cell killing via Natural Killer group 2, member D (NKG2D) mechanisms, the mechanism of action of bortezomib, its relatively short half-life, and its ability to be administered systemically may be more attractive and efficacious as a strategy to augment NK function in a clinical setting.

In this report, we show that ALDH^bright^ CSCs from several solid tumor types, including human primary tumors are sensitive to induction of NK ligands MICA and MICB following bortezomib treatment. Additionally, we show that bortezomib treatment leads to an enrichment of ALDH^bright^ cells. Moreover, bortezomib pre-treatment significantly sensitizes ALDH^bright^ cells to NK cell killing both in vitro and in vivo, leading to improved elimination of CSCs and significant tumor growth delay. Together, these results suggest that this immuno-oncology combination using NK cells may prove useful as a mean to target CSCs and improve oncologic outcomes in otherwise refractory malignancies.

## 2. Results

### 2.1. Bortezomib Treatment Enriches for ALDH^bright^ Tumor Cells from Several Solid Tumor Types

**Figure 1:**
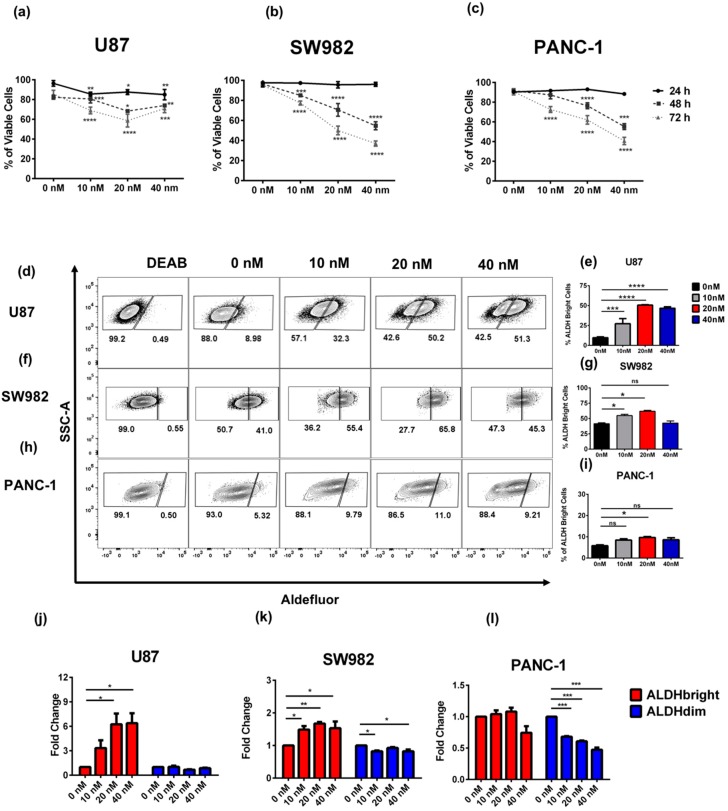
Dosing effect of bortezomib on ALDH^bright^ cells. The anti-viability effects of bortezomib treatment were assessed in glioblastoma (U87) (**a**), synovial sarcoma (SW982) (**b**), and pancreatic adenocarcinoma (PANC-1) (**c**) cells. U87 (**d**,**e**), SW982 (**f**,**g**), and PANC-1 (**h**,**i**) cells were analyzed by flow cytometry after 48 h of bortezomib for the frequency of live cells with an ALDH^bright^ phenotype. The fold change of viable ALDH^bright^ and ALDH^dim^ cells was compared using flow cytometry for U87 (**j**), SW982 (**k**), and PANC-1 (**l**) cell lines. DEAB: Representative gating for the N,N-diethylaminobenzaldehyde control selective inhibitor of aldehyde dehydrogenase isoenzymes. Experiments were repeated three-five times, including a minimum of three technical replicates for each experiment. (* = *p* < 0.05, ** = *p* < 0.01, *** = *p* < 0.001, **** = *p* < 0.0001).

Given our experience using radiotherapy as a way to enrich for stem-like tumor cells, we first sought to evaluate the impact of bortezomib exposure on the ALDH phenotype of tumor cells in vitro. Following 24, 48, or 72 h incubation, we evaluated cytotoxic effects of bortezomib on human U87 (glioblastoma), SW982 (synovial sarcoma), and PANC-1 (pancreatic adenocarcinoma) cell lines. We observed a similar decrease in cell viability in a dose and time dependent manner in SW982 and PANC-1 cell lines, while U87 showed a modest resistance to the cytotoxic effects of bortezomib (Figure 1a–c and Figure S1a–f). Based on these observations, we then decided to assess ALDH expression following a 48-h incubation in media containing 0, 10, 20, and 40 nM of bortezomib. In these experiments (Figure 1d–i), we observed significant enrichment in ALDH^bright^ cells, particularly in U87 and SW982.

We then sought to determine if the enrichment in ALDH^bright^ cells following bortezomib treatment was due to direct effects of bortezomib on the ALDH^bright^ population, due to effects on the ALDH^dim^ population, or both. Following incubation with bortezomib for 48 h, cells were analyzed by flow cytometry for the frequency and number of ALDH^bright^ and ALDH^dim^ cells in culture (Figure S1g–l). In these experiments, we observed a differential response of ALDH^bright^ and ALDH^dim^ cells to bortezomib treatment. For instance, in U87 cells, we observed increased frequency and numbers of ALDH^bright^ cells (Figure 1d,e,j and Figure S1g,j). We observed similar effects in SW982 cells with a significant increase in ALDH^bright^ numbers in ALDH^bright^ cells at both 10 and 20 nM concentrations, respectively, and by frequency at 10, 20, and 40 nM (Figure 1f,g,k and Figure S1h,k). In SW982 cells, we also noted a modest decrease in the frequency of the ALDH^dim^ population following bortezomib treatment (Figure 1k and Figure S1k). In contrast, the PANC-1 cell line (Figure 1h,i,l) showed an increase in frequency in the ALDH^bright^ subpopulation across all treatment conditions; however, these differences were only significant at 20nM of bortezomib. In the PANC-1 cell line, we did not observe an increase by numbers in the ALDH^bright^ subpopulation (Figure S1i). However, we observed a dose response represented by a fold change decrease in the fraction of ALDH^dim^ cells relative to ALDH^bright^ cells in PANC-1(Figure 1l) and a decrease by cell number for this particular subpopulation (Figure S1l). Interestingly, even though we observed ALDH enrichment effect across the different cell lines tested, the 20 nM concentration seemed to be the optimal concentration where ALDH enrichment occurred while at 40 nM of bortezomib greater anti-viability effects occurred in both sub-populations. Taken together, these data suggest that the mechanism of ALDH^bright^ enrichment is the result of a greater resistance to the cytotoxic/cytostatic effects of bortezomib among ALDH^bright^ versus ALDH^dim^ cells across cancer cell lines.

### 2.2. Bortezomib Increases the Expression of Stress Ligands and Death Receptors on both ALDH^bright^ and ALDH^dim^ Cells

**Figure 2:**
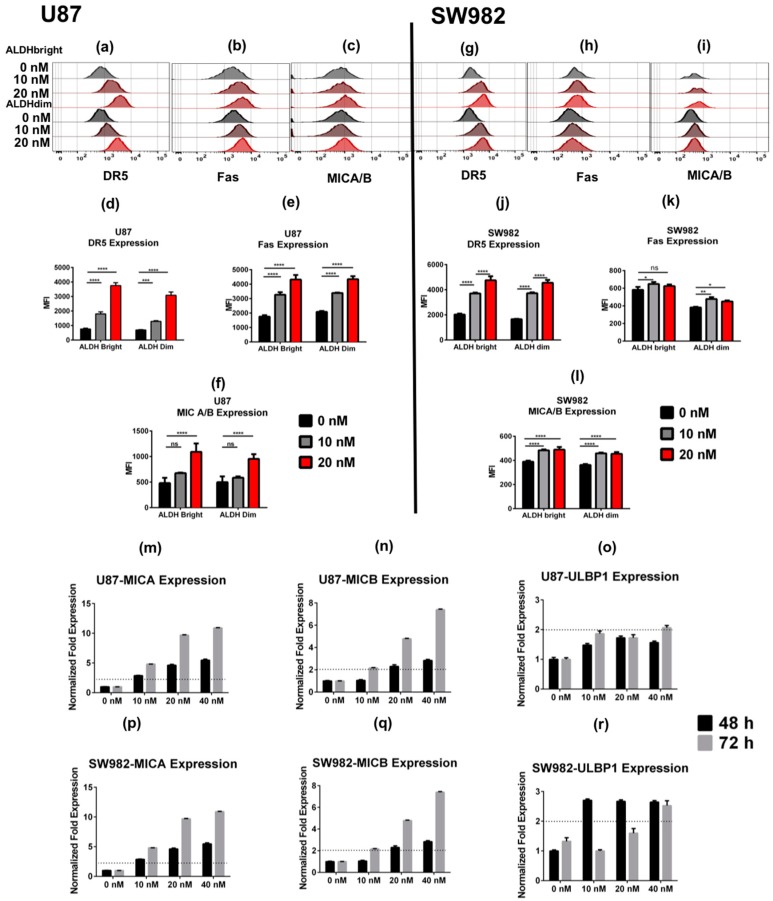
Bortezomib induces expression of death receptors and natural killer (NK) stress ligands. U87 (**a**–**f**) and SW982 (**g**–**l**) cells were assessed by flow cytometry for the expression of death ligands and stress ligands in U87 and SW982 cells stratified by ALDH^bright^ and ALDH^dim^ expression. Overlaid histograms are representative for the expression of DR5, Fas, and MICA/B. Bar graphs were generated from median fluorescence intensity (MFI) calculations for each stated condition comparing the MFI from ALDH^brigh^ and ALDH^dim^ populations in each of the treatments, respectively. Expression of *MICA*, *MICB*, and *ULBP1* in U87 (**m**–**o**) and SW982 cells (**p**–**r**) was assessed by qPCR after 48 and 72 h of bortezomib treatment, respectively. Dotted lines indicate a two-fold expression threshold consistent with significant upregulation. Experiments were repeated three–five times including a minimum of three technical replicates for each testing condition. (* = *p* < 0.05, ** = *p* < 0.01, *** = *p* < 0.001, **** = *p* < 0.0001).

Bortezomib has been shown to induce the expression of death receptors such as DR5 on the surface of both mouse and human tumor cell lines [26]. Therefore, we next evaluated if bortezomib would induce differential expression of death receptors and stress ligands on ALDH subpopulations in our cancer cell lines. Bortezomib significantly upregulated the expression of DR5, Fas, and MICA/B on both ALDH^bright^ and ALDH^dim^ U87 cells in vitro (Figure 2a–f). Similarly, we observed a significant increase in DR5, MICA/B, and Fas expression in SW982 cells following bortezomib treatment (Figure 2g–l). For each protein examined, bortezomib induced a dose-dependent increase in protein expression with 20 nM of bortezomib showing the highest level of upregulation as quantified by median fluorescence intensity (MFI) level by flow cytometry. Additionally, we compared the mRNA expression of *MICA*, *MICB*, and *ULBP1* in U87 and SW982 cells after 48 and 72 h of bortezomib exposure (Figure 2m–o). U87 cells increased expression of the both *MICA* and *MICB* at 48 h and 72 h post-treatment. However, we observed *ULBP1* to be more than two-fold upregulated only at a dose of 40 nM at 72 h post-treatment (Figure 2o). In SW982 cells, we observed a similar upregulation of *MICA* and *MICB* gene expression at both 48 and 72 h time points post-treatment (Figure 2p,q). Interestingly, the expression of *ULBP1* increased by at least two-fold in SW982 cells at 48 h at doses of 10 and 20 nM, however, gene expression levels decreased at 72 h. In the 40 nM treatment group, the expression was a consistent significant increase in *ULBP1* expression >2-fold baseline at both 48 and 72 h. Given reports that bortezomib treatment decreases MHC class I expression in ALDH^bright^ cells in multiple myeloma and thereby sensitizes myeloma to NK killing [27], we then investigated the expression of MHC class I in our glioblastoma and sarcoma cancer lines after bortezomib exposure. Although we observed increased expression of MHC class I in U87 cells treated at 10 and 20 nM doses for 48 h with exception between 0 and 10 nM in the ALDH^dim^ subpopulation (Figure S2a), no significant differences were observed in SW982 (Figure S2b). It has been reported previously that the absence or downregulation of MHC class I molecules by tumor cells could be associated with escape mechanisms from T cell targeting [28]. In this regard, upregulation of MHC class I expression on U87 cells and maintenance on SW982 cells may represent an inducible pathway that cancer cells utilize to resist immune pressure from NK or T cells depending on the dominant cellular/local conditions. Regardless, our data demonstrate that upregulation of NK ligands is a prevalent phenomenon among tumor cells after bortezomib exposure. Although this upregulation occurred in both ALDH^bright^ and ALDH^dim^ subsets, the increase in ALDH^bright^ cell numbers and frequencies after bortezomib exposure suggests that expression of these stress ligands is relevant to NK targeting of these resistant cells after cytotoxic/cytostatic drug therapies.

### 2.3. U87 ALDH^bright^ Subpopulations Exhibit Increased Expression of Cancer Stem Cell-Related Markers Compared to ALDH^dim^ Subpopulations

**Figure 3:**
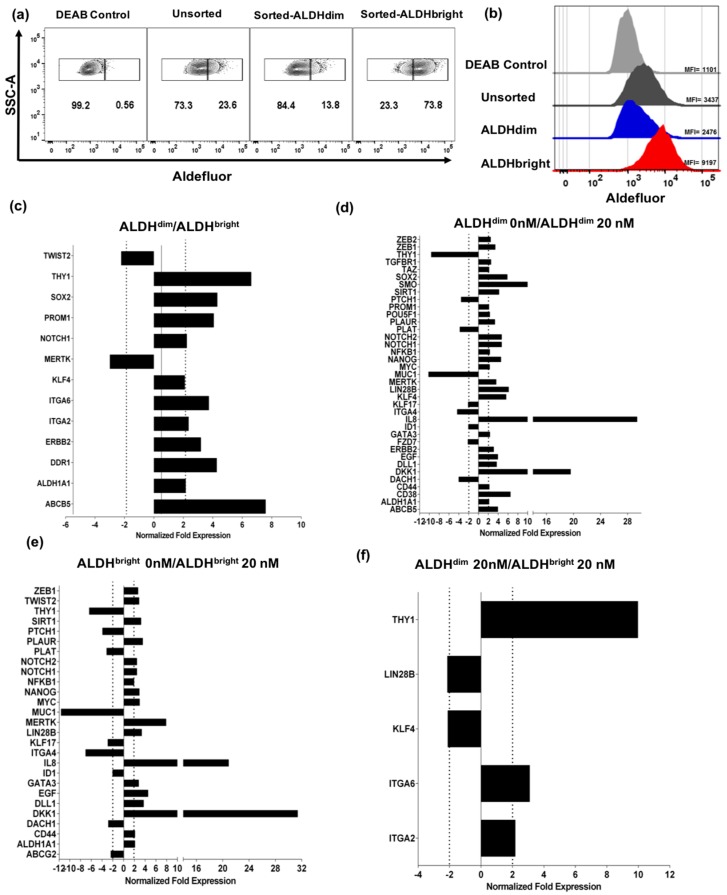
Bortezomib increases expression of cancer stemness genes in sorted ALDH^bright^ and ALDH^dim^ subpopulations. (**a**) Flow cytometry plots show frequencies of ALDH^bright^ and ALDH^dim^ subpopulations after sorting and expansion compared to unsorted parent control. (**b**) Histograms show increased expression of ALDH by MFI in enriched ALDH^bright^ sorted cells and decreased expression of ALDH by MFI on sorted ALDH^dim^ cells compared to unsorted cells and DEAB control. Bar graphs generated from qPCR arrays show differential expression of cancer stemness-associated markers in sorted subpopulations. (**c**) ALDH^dim^ compared to ALDH^bright^ untreated cells, (**d**) ALDH^dim^ untreated (0 nM) compared to ALDH^dim^ treated with 20 nM bortezomib, (**e**) ALDH^bright^ untreated (0 nM) compared to ALDH^bright^ treated with 20 nM bortezomib, (**f**) ALDH^dim^ treated compared to ALDH^bright^ treated with 20 nM bortezomib. Dotted lines indicate a two-fold expression threshold consistent with significant upregulation.

Next, we sought to investigate gene signature differences between the ALDH^bright^ and ALDH^dim^ sorted tumor subpopulations. Therefore, we sorted, then expanded and phenotyped ALDH^bright^ and ALDH^dim^ cells from U87 parent cells. We observed that after short term culture over 1–2 weeks, we were able to maintain relatively pure ALDH^bright^ and ALDH^dim^ populations (Figure 3a,b). The frequency of the ALDH^dim^ subpopulation in the sorted and expanded ALDH^dim^ subpopulation remained at approximately 14% with an MFI of 2537 ± 125, while in the ALDH^bright^ population the frequency of ALDH^bright^ cells remained at approximately 74% with an MFI of 8570 ± 545. In the unsorted parent cells, the population frequency of the ALDH^bright^ population was approximately 24% with an MFI of 3195 ± 18 (Figure 3a,b and Figure S3a). Using these sorted and short-term expanded ALDH subpopulations, we then isolated RNA and performed a PCR array, analyzing 88 unique target genes associated with cancer stem cell signatures. After analysis, we observed that the ALDH^bright^ subpopulation demonstrated increased expression of *ABCB5, ALDH1A1, DDR1, ERBB2, ITGA2, ITGA6, KLF4, NOTCH1, PROM1, SOX2,* and *THY1* genes. In contrast, expression of *MERTK1*, and *TWIST2* was found to be downregulated (Figure 3c). Interestingly, the majority of the genes that we found to be upregulated in the ALDH^bright^ subpopulation have been reported as markers involved not only in CSCs in general, but particularly in brain tumor CSCs [29,30,31].

#### Bortezomib Affects Expression of Cancer Stem Cell-Related Genes and MICA/B in both ALDH^bright^ and ALDH^dim^ Subpopulations

We then evaluated the effect of bortezomib on sorted and expanded populations after 48 h of bortezomib at 20 nM compared to untreated controls (0 nM). When comparing the untreated ALDH^dim^ subpopulation to 20 nM treated cells, we observed an increase in expression of *SOX2, ALDH1A1, DKK1, KLF4, PROM1, ERBB2,* and *ABCB5* among others. Additionally, we observed a downregulation of THY1 and *MUC1* among others (Figure 3d). When comparing treated to untreated ALDH^bright^ cells, we observed an upregulation of *ALDH1A1*, *CD44, IL8, DKK1, EGF*, *MERTK*, *MYC*, *NANOG*, *NFKB1*, *TWIST2*, *ZEB1* and downregulation of *THY1* and *MUC1* (Figure 3e). When comparing treated ALDH^bright^ to ALDH^dim^ populations, we observed an upregulation of *THY1, ITGA2*, and *ITGA6* and a downregulation of *KLF4* and *LIN28B* (Figure 3f). Interestingly, these data suggest that *THY1* and *ITGA6* are likely directly associated with ALDH expression independent of treatment while *ALDH1A1*, *IL8*, and *DKK1* are potentially upregulated in response to bortezomib treatment in both ALDH^bright^ and ALDH^dim^ subpopulations. It is important to note that these results may be further clarified by improving the sorting purity of both ALDH^bright^ and ALDH^dim^ subpopulations, as certain gene signatures may be affected by residual ALDH^bright^ cells in the ALDH^dim^ enriched subpopulation (extended gene analysis results for this particular assay are shown in Figure S3f). Of note, we observed increased *ALDH1A1* expression in response to bortezomib treatment in both ALDH^bright^ and ALDH^dim^ subpopulations similar to *ALDH2* (Figure S3b,c). This particular finding may suggest that not only is *ALDH1A1* upregulated in response to bortezomib, but also other gene members of the aldehyde dehydrogenase superfamily such as *ALDH2*. Additionally, we analyzed the expression of *MICA* and *MICB* in response to bortezomib, observing that both ALDH^bright^ and ALDH^dim^ populations exhibited upregulation of NKG2D ligands (Figure S3d,e). These data agree with our previous findings in unsorted cells where both subpopulations showed increased expression of *MICA* and *MICB*. The biological significance of NKG2D ligand upregulation in both ALDH^bright^ and ALDH^dim^ subsets warrants further exploration, although our data suggest that ALDH^dim^ cells are eliminated at higher rates by NK cells compared to ALDH^bright^ cells in response to bortezomib treatment. This may be unrelated to expression of NK ligands; however, it may also represent the effects of differential shedding of NK ligands by ALDH^dim^ subsets via ADAM17 and other proteases.

### 2.4. Bortezomib Enriches for ALDH^bright^ Cells from Human Primary Glioblastoma and Breast Tumor

**Figure 4:**
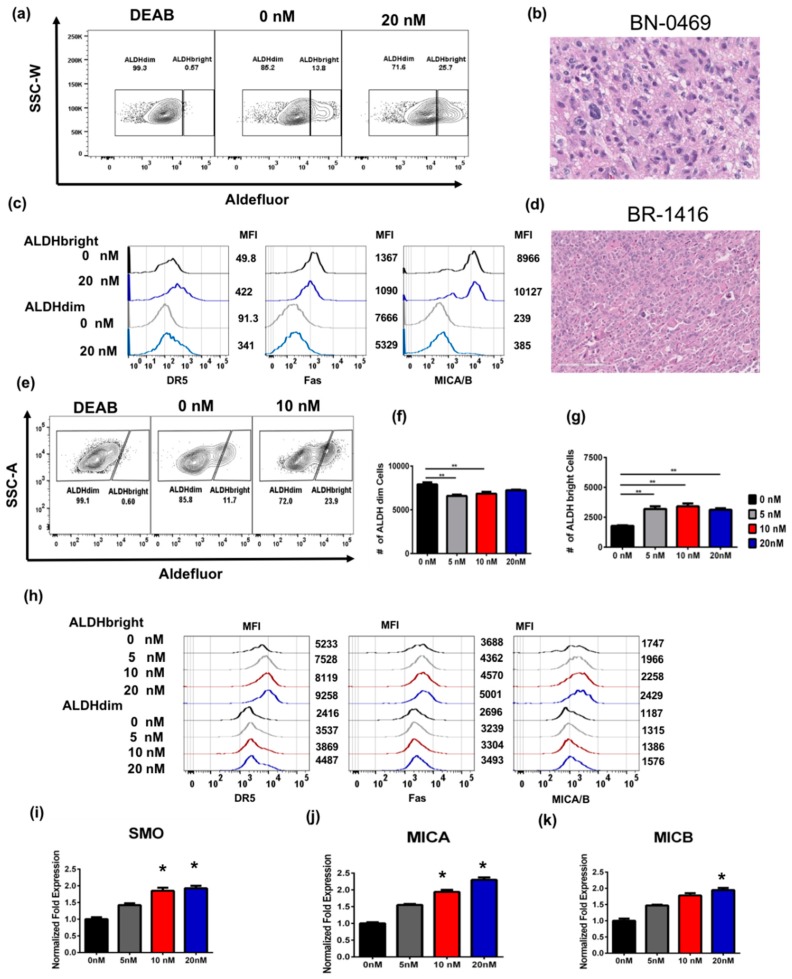
Effects of bortezomib on primary human brain and breast cancer cells. Freshly excised primary glioblastoma multiforme (BN-0469) and primary breast cancer (BR-1416) surgical specimens were treated ex vivo with increasing doses of bortezomib. (**a**) After overnight incubation with 20 nM bortezomib, BN-0469 showed significant upregulation of ALDH^bright^ cells when compared to both DEAB and vehicle controls. (**b**) Representative hematoxylin and eosin staining (H&E) section of BN-0469 shows characteristic features of glioblastoma multiforme, 20× magnification. (**c**) Representative histogram for BN-0469 treated with 0 nM (black histogram representative for ALDH^bright^ and gray histogram representative of ALDH^dim^ subpopulations, respectively) or 20 nM (dark blue histogram representative of ALDH^bright^ and light blue for ALDH^dim^ subpopulations, respectively). MFI values are adjacent to the corresponding histograms. (**d**) Representative histopathology of high grade, mitotically active breast carcinoma, 10× magnification. (**e**) After 24 h treatment with either 5, 10, or 20 nM bortezomib treatment, there was a significant increase in frequency of ALDH^bright^ cells on all bortezomib treated cells ex vivo. (**f**,**g**) Bar graph shows the number of ALDH^bright^ and ALDH^dim^ cells in BR-1416 cells after bortezomib for 24 h. (h) Representative histogram shows the expression by MFI of DR5, Fas and MICA/B in ALDH^bright^ and ALDH^dim^ on BN-0469. (**i**–**k**) qPCR of BR-1416 cells treated with bortezomib after 24 h shows a significant dose dependent increase in expression of Smoothened Frizzled Class Receptor (*SMO)*, *MICA*, and *MICB*. (* = *p* < 0.05, ** = *p* < 0.01, *** = *p* < 0.001).

Next, we assessed the effects of bortezomib on primary human tumor samples as the use of continually passaged cell lines has been called into question, particularly in studies of the genetic and epigenetic factors which impact tumor heterogeneity and CSC behavior. Therefore, to better validate the relevance of our findings, we assessed freshly excised tumor samples from patients undergoing surgical resection. We analyzed a human primary glioblastoma tumor and a human primary breast cancer, which were mechanically and enzymatically dissociated and then plated in standard cell culture medium as a single cell suspension for 24 h. Treatment of tumor cells from a primary brain tumor with 20 nM bortezomib led to a significant increase in the proportion of ALDH^bright^ cells (Figure 4a,b). Additionally, we observed a significant increase in the expression by MFI of DR5, and MICA/B, but not Fas as previously observed with the U87 cell line (Figure 4c). Similar results were observed with tumor cells from a primary breast cancer surgically removed and then treated with 0, 5, 10, and 20 nM bortezomib ex vivo after single cell digestion (Figure 4d–g). In this patient-derived specimen, we observed an increase in the number of ALDH^bright^ cells when the cells were treated with bortezomib compared to the untreated control (Figure 4f). We observed a significant decrease in cell numbers on the ALDH^dim^-treated cells at 5 and 10 nM of bortezomib (Figure 4f). Flow cytometric analysis revealed that DR5, Fas and MICA/B expression was increased on ALDH^bright^ cells after bortezomib treatment (Figure 4h), although effects were observed in ALDH^dim^ cells as well. Additionally, we observed an increase in the mRNA expression levels of Smoothened Frizzled Class Receptor (*SMO*), a hedgehog transducer that has been previously associated with tumorigenesis in a dose dependent manner (Figure 4i) [32]. We then evaluated the expression levels of *MICA* (Figure 4j) and *MICB* (Figure 4k) genes. We found that *MICA* was significantly upregulated in cells treated with 10 and 20nM of bortezomib while *MICB* was significantly upregulated at 20 nM of bortezomib. Taken together, these data suggest that enrichment of ALDH^bright^ stem-like tumor cells and the simultaneous increase in the cell surface expression of DR5, MICA, and MICB following bortezomib treatment occurs in primary patient-derived samples in addition to immortal cancer cell lines grown in adherent cell culture conditions. Given the increasing attention to the spontaneous tumors in outbred dogs as a model to facilitate translation of novel immunotherapy approaches [33,34], we also evaluated primary dog sarcomas treated ex vivo with bortezomib. Similar to human spontaneous tumors, we observed that bortezomib pre-treatment enriched for both the numbers and frequencies of ALDH^bright^ tumor cells and that bortezomib demonstrated increasing anti-viability effects at doses up to 40 nM, leading to enrichment in the number of ALDH^bright^ CSCs (Figure S4).

### 2.5. ALDH^bright^ Cells Are Sensitized to NK Cell Killing following Bortezomib Pre-Treatment In Vitro

**Figure 5:**
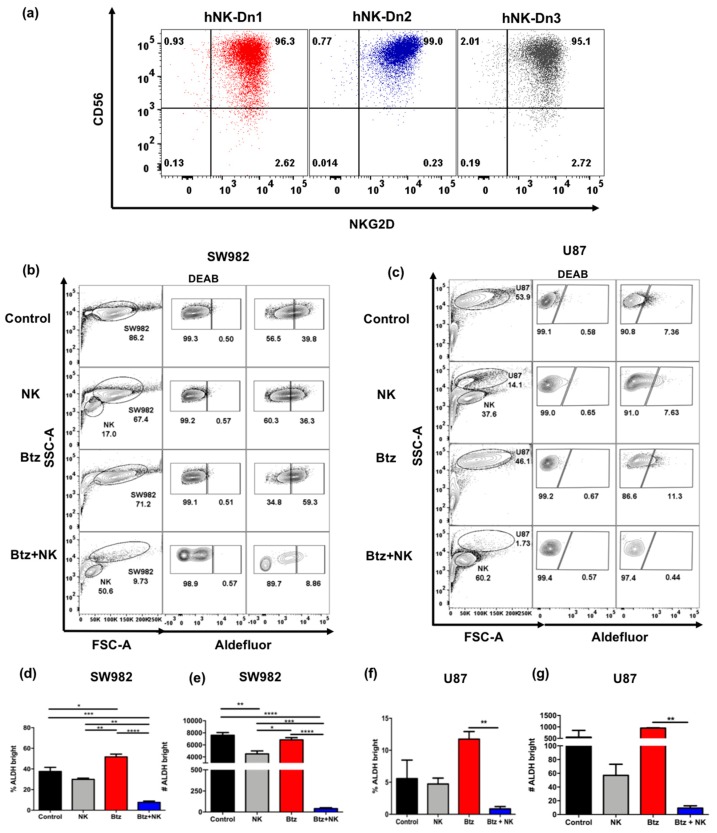
Bortezomib promotes NK cell killing of ALDH^bright^ cells in vitro. (**a**) Representative flow cytometry shows CD56 and NKG2D expression on expanded and activated human NK cells from three different donors (hNK-Dn1- hNK-Dn3) after 14 days in co-culture with irradiated K562 feeder cell line and 100 IU/mL rhIL-2. (**b**) SW982 and (**c**) U87 cells were pretreated with or without 20 nM bortezomib for 24 h prior to co-culture with activated human NK cells at an Effector: Target (E:T) ratio of 0.125:1 for 12 h. Representative flow cytometry showed increased NK killing of ALDH^bright^ cancer cells after 20 nM bortezomib. The frequency and number of ALDH^bright^ SW982 (**d**,**e**) and U87 (**f**,**g**) cells in different co-culture conditions were assessed. (* = *p* < 0.05, ** = *p* < 0.01, *** = *p* < 0.001, **** = *p* < 0.0001).

Based on our previous work demonstrating that NK cells have the ability to lyse stem-like tumor cells, including ALDH^bright^ cells in vitro and in vivo, we next assessed whether NK cytotoxicity against ALDH^bright^ cells would be augmented following bortezomib pretreatment. First, we expanded and activated human NK cells extracted from human peripheral blood mononuclear cells (PBMC) followed by activation and expansion for 14–21 days. After 14 days in culture, we evaluated the phenotype of expanded NK cells and found comparable expression of CD56 and NKG2D in NK cells expanded from three different donors (Figure 5a). SW982 sarcoma cells were pretreated with medium containing 0 or 20 nM concentration of bortezomib for 24 h. Following bortezomib treatment, cells were washed with fresh medium and then co-cultured with activated allogeneic NK cells at an effector to target ratio of 0.125:1. After bortezomib pretreatment and NK co-culture, we observed a significant reduction in the frequency of ALDH^bright^ cells (Figure 5b,d). In addition to the reduction in ALDH^bright^ frequency, we observed reductions in the total number of viable ALDH^bright^ cells after bortezomib and NK co-culture (Figure 5e).

We then assessed the effects of bortezomib-mediated sensitization to NK cell killing on U87 cells and observed a similar pattern of enhanced cytotoxicity (Figure 5c,f). In fact, the combination of bortezomib pretreatment and NK cell exposure led to a dramatic and statistically significant reduction in the frequency of ALDH^bright^ cells remaining after 24 h (Figure 5f), as well as a reduction in the overall numbers of ALDH^bright^ cells (Figure 5g). Therefore, we concluded that bortezomib is not only capable of enriching for ALDH^bright^ stem-like tumor cells, but also sensitizes those cells to NK cell cytotoxicity, presumably due to the bortezomib-induced enhanced expression of NK ligands.

### 2.6. The Combination of NK Cells and Bortezomib Leads to Reduced Stem-Like Tumor Cells In Vivo and Promotes Tumor Regression

**Figure 6:**
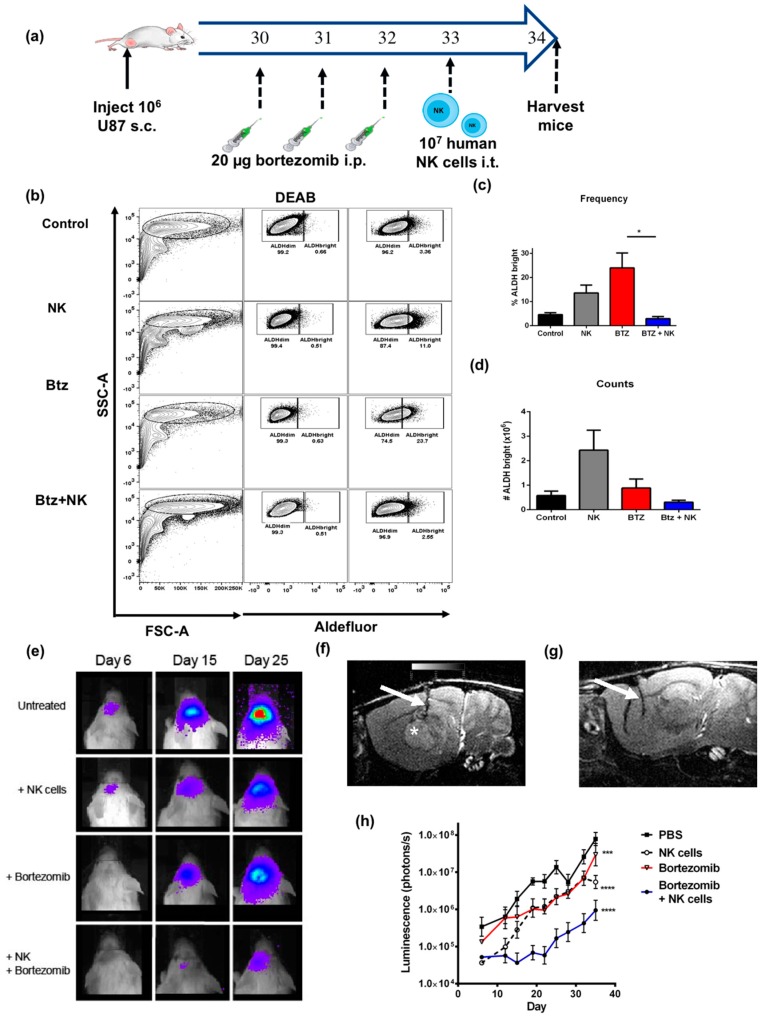
Bortezomib promotes NK cell killing of ALDH^bright^ cells in vivo and augments NK anti-tumor effects. (**a**) Schema depicts experimental design for in vivo treatment of NOD scid gamma mice (NSG) mice implanted subcutaneously with U87 cells. Tumors were then allowed to grow for 30 days before bortezomib treatment and NK transfer. (**b**) Representative flow cytometry plots demonstrate the tumor cell population with gating on CD45 positive and 7-aminoactinomycin D (7-AAD) viable tumor cells. (**c**) Bar graph quantifies (*n* = 3 mice/group) ALDH^bright^ cells in vivo after bortezomib treatment compared to controls. (**d**) Bar graph shows the number of ALDH^bright^ cells recovered in vivo among the different treatment groups. Luciferase transfected U87 cells were orthotopically implanted in the brain of NSG mice and subsequently treated with bortezomib and/or NK cells. Animals were imaged twice weekly to assess tumor burden based on bioluminescence. (**e**) Representative bioluminescent images of mice are shown for each time point. Representative MRI images of xenografts from an untreated mouse (**f**), and bortezomib and NK cell treated mouse (**g**) at day 25 are also shown with evidence for notable decrease in tumor size post combination treatment. The needle tract for intra-tumoral NK injection is marked by the arrow and the tumor is marked by the white star, which is nearly completed resolved. (**h**) We assessed the tumor burden of orthotopic U87 xenografts by bioluminescence over time (*n* = 3 mice/group). (* = *p* < 0.05, ** = *p* < 0.01, *** = *p* < 0.001, **** = *p* < 0.0001).

Based on these data, we then sought to assess the efficacy of bortezomib plus NK adoptive transfer in vivo. First, we tested if the augmented ALDH^bright^ tumor cell killing we observed in vitro could be replicated in vivo, where the three-dimensional (3D) nature of the tumor and issues of drug and NK cell accessibility to the tumor could alter the effectiveness of either treatment alone or the combination. To do so, we implanted subcutaneous U87 tumors into the flanks of immunodeficient NSG mice (Figure 6a). When tumors reached 5–7 mm in diameter, mice were injected with 20 μg of intraperitoneal (i.p.) bortezomib ×3 and/or activated NK cells, and tumors were assessed for changes in ALDH^bright^ cells following in vivo treatment (Figure 6b). Similar to the results observed in vitro, bortezomib alone led to a significant increase in the frequency of ALDH^bright^ cells (Figure 6c), with little effect on the total number of ALDH^bright^ cells (Figure 6d) within the tumor. In contrast, the combination of bortezomib and NK cells administered intra-tumorally 24 h after bortezomib treatment led to a significant decrease in both the frequency and total number of ALDH^bright^ cells recovered (Figure 6c,d).

Next, we assessed the therapeutic potential of this combination therapy in NSG mice bearing orthotopic intracranial U87 xenografts. Briefly, luciferase transfected tumor cells were implanted into the brain using stereotactically guided instrumentation. Following tumor implantation, mice were treated with i.p. bortezomib as before. Thereafter, a single injection of 10^6^ activated allogeneic human activated NK cells were administered intratumorally. Tumor size was monitored via bioluminescence, and quantification was assessed starting from the date of bortezomib treatment. Importantly, the administration of either NK cells alone or bortezomib alone had a noticeable effect on tumor size six days after treatment (Figure 6e). However, by 25 days after treatment, the anti-tumor effects of bortezomib monotherapy and NK cell monotherapy, but not combination therapy, were lost. Mice that received the combination of bortezomib and NK cells demonstrated a significant reduction in tumor size, which persisted to day 25 (Figure 6g), compared to untreated mice (Figure 6f). Additionally, the overall tumor growth was significantly delayed for the combination therapy group as compared to all other groups. We assessed the tumor burden of orthotopic U87 xenografts by bioluminescence over time showing significantly less bioluminescence in the bortezomib plus NK treated mice at all time points starting at day 15 (Figure 6h). These results suggest that the combination therapy could act to reduce tumor growth over time by limiting the potential for ALDH^bright^ stem-like tumor cells to continue to seed the tumor niche with non stem-like cells.

## 3. Discussion

In these studies, we demonstrated the presence of a subpopulation of ALDH^bright^, stem-like tumor cells, which appear relatively resistant to the direct cytotoxic effects of the proteasome inhibitor bortezomib, but demonstrate upregulation of NK ligands and death receptors, which allow for additive anti-tumor effects when bortezomib pre-treatment is followed by NK immunotherapy in vitro and in vivo. Notably, the ALDH^bright^ cells, which survived following bortezomib treatment, appeared to become sensitized to NK cell mediated killing through the concomitant upregulation of surface molecules, which facilitated NK cell mediated cytotoxicity, such as MICA/B, DR5, and Fas.

While other groups have demonstrated that the combination of bortezomib and NK cells is effective in pre-clinical studies, our study is one of the first to highlight the ability of this combination to target CSCs, especially in solid tumor models. In addition, we assessed the anti-tumor effects in vivo, which provide further support of the potential for this combination to be clinically meaningful. Although the combination is under investigation in clinical trials (primarily in the post bone marrow transplantation scenario), it is important to recognize the impact of the dosing and timing of both treatments on the potential success of this strategy. We have previously demonstrated that bortezomib is cytotoxic to NK cells in vitro, and as such, the timing of this combination therapy must be carefully planned to avoid bortezomib-mediated NK cell inhibition in vivo [17,35]. Additionally, in our in vivo model, NK cells were administered directly into the tumor site following systemic bortezomib administration. One of the major limitations to the efficacy of adoptive NK cell therapy is the limited tumor-infiltrating capacity that is observed with NK cell adoptive transfer in vivo. The use of an intralesional injection strategy aims to circumvent this limitation, allowing highly activated NK cells to interact directly in the tumor niche; however, this strategy may not be widely applicable in the setting of diffuse metastatic disease. For this reason, glioblastoma and other locally aggressive tumors may be particularly suited to NK immunotherapy strategies, since intra-tumoral delivery of NK cells is more feasible in these situations.

Yet, despite the efficacy observed in our model system, several caveats must be considered before this approach can be successfully translated to the clinic. First, it is unclear if and how CSC selection could occur following this therapy. It is possible that within the CSC niche, subsets of CSCs exist that could evade NK cell detection, either by harboring an inherent resistance to the sensitizing effects of bortezomib, or by altering the expression of various factors that could regulate NK cell responses. Indeed, many of the tumor-NK cell escape mechanisms that have been proposed could also be applicable to CSCs, such as heightened secretions of TGF-β and IL-10 [36,37,38,39,40].

Additionally, it remains to be determined how and why ALDH^bright^ cells can resist the apoptotic effects of bortezomib. Previous studies suggested that the Wnt/β-catenin signaling pathway can stimulate tumor cells with resistance mechanisms against bortezomib. Indeed, bortezomib has been found to lead to increased β-catenin accumulation in tumor cells, and β-catenin has separately been shown to drive the expression of ALDH in radio-resistant prostate cancer cells [41,42]. Therefore, increased expression of β-catenin as a response to bortezomib treatment may simultaneously stimulate ALDH expression in CSCs and promote resistance. Interestingly, previous studies have also found that the selective ALDH inhibitor, disulfiram, can overcome bortezomib resistance in AML cells [2]. Similarly, in proliferating, sub-confluent, endothelial cell cultures, it has been shown that proteasome inhibition can induce apoptosis at a concentration 340-fold lower than that required in quiescent cells, suggesting that proteasome inhibition may affect tumor growth by preferentially inhibiting proliferating cells/non-CSCs [43]. In a related publication, the same group demonstrated that proteasome inhibition induced apoptosis in proliferating cells while showing evidence of protective effects in some quiescent cells [44]. Increased ALDH levels in CSCs may have a direct effect in resistance to the cytotoxic effects of bortezomib, especially as cellular proliferation versus quiescence is considered a cardinal feature in distinguishing CSCs from non-CSCs [15,16]. Additionally, our studies suggest that the ALDH^bright^ population validates the CSC phenotype based on the increased expression of brain cancer stem cells genes such as *ALDH1A1*, *ITGA6*, *THY1*, *PROM1*, and *SOX2,* as reported previously. Moreover, our studies showed an increase in the expression of multiple other genes associated with CSC phenotype in U87 cells following bortezomib treatment, including *IL8, DKK1*, and *SMO,* which have all been reported to be involved in stemness pathways as well as increased drug resistance [29,30,31].

Ultimately, while the mechanisms of ALDH-mediated resistance mechanisms remain unclear, our in vitro and in vivo data suggest that the combination of bortezomib and NK cell therapy may represent an attractive strategy to target residual CSCs that may persist and/or seed relapse in a variety of solid malignancies after standard cytotoxic treatments. It is important to consider potential barriers that may arise while using autologous or allogeneic derived, expanded, and activated human NK cells. Some of the limitations may include hyporesponsiveness and survival of NK cells when adoptively transferred into cancer patients due to the notable change from an in vitro environment with maximal cytokine stimulation to an in vivo environment less replete in cytokine support. To maintain infused NK cells in an activated state in vivo in our models, we injected an IL-15-producing plasmid as described in our prior publications. Although clinical experience in human cancer patients has shown favorable responses with intravenous rhIL-2, concerns exist regarding the potential immunosuppressive effects of rhIL-2, including induction of regulatory T cells (Tregs) and activation-induced cell death of immune effector cells. In contrast, likely because of the trans-presentation of IL-15:IL-15Rα complexes rather than solubilization of the cytokine for signaling, IL-15 has negligible if any effects on Tregs and provides anti-apoptotic signals to CD8+ T cells. Therefore, there is increasing attention on strategies combining IL-15 and NK immunotherapy to maximize anti-tumor effects and limit NK hyporesponsive following adoptive transfer. However, other causes of NK cell hyporesponsiveness after in vivo transfer include inhibitory signaling produced by the tumor microenvironment, such as increased production of inhibitory cytokines, deficient recruitment into the tumor site, and deficient migration and/or engraftment of NK cells into the tumor [45]. In order to overcome some of these limitations, some investigators have proposed a selection of expanded NK cell subpopulations expressing CXCR3, which increased migration toward solid tumors or genetic overexpression of CXCR2 to promote tumor homing as has been demonstrated in a renal cell carcinoma model [46,47,48].

Ultimately, further studies are needed to understand intrinsic resistance mechanisms, routes of NK administration, and in vivo activation strategies in order to optimally apply NK cells as a cancer therapeutic. Combination therapy with proteasome inhibitors such as bortezomib may be one such strategy given the CSC targeting effects.

## 4. Materials and Methods

### 4.1. Tumor Cell Lines

U87MG, PANC-1 and SW982 were purchased from American Type Culture Collection (ATCC, Manassas, VA, USA) and propagated in the culture medium recommended by ATCC. Fresh de-identified primary human tumor samples (BN-0469 primary glioblastoma and BC-1154 primary breast cancer) were obtained immediately following surgical resection from the UC Davis Comprehensive Cancer Center Biorepository. Human tumors were mechanically and enzymatically dissociated using a 60-min incubation of 1 mg/mL collagenase-IV and 0.1 mg/mL DNAse I at 37 °C. For all experiments, primary cells were used within 48 h of isolation. Consent was obtained from all patients before tissue procurement under the auspices of the Institutional Review Board of UC Davis (Protocol # 218204).

### 4.2. NK Cell Isolation

Human NK cells were isolated from leukoreduction system (LRS) chambers obtained from healthy donors (BloodSource, Sacramento, CA, USA). LRS chambers were back-flushed with PBS and lymphocytes were isolated following high-speed centrifugation with Lymphocyte Separation Medium (Cellgro, Manassas, VA, USA) as per manufacturer’s instructions. Lymphocytes were then counted using a Z Series Coulter Counter (Beckman Coulter, Indianapolis, IN, USA) and re-suspended at 5 × 10^7^ cells/mL with an additional 1000 patient-matched red blood cells per lymphocyte. NK cells were isolated from the above samples using a RosetteSep™ Human NK Cell Enrichment Cocktail (STEMCELL Technologies, Vancouver, BC, Canada) as per manufacturer’s instructions. This protocol typically yielded >95% CD45+/CD56+/CD3− cells.

### 4.3. NK Cell Expansion and Activation

The artificial antigen presenting cell line K562 clone 9.mbIL-21 was used as a feeder cell source to promote expansion and activation of primary human NK cells in vitro. These cells were kindly provided by Dr. Dean Lee (Nationwide Children’s Hospital, Columbus, OH, USA). Freshly isolated NK cells were co-cultured with K562 clone 9.mbIL-21 cells as previously described [49]. Briefly, freshly isolated NK cells were mixed with irradiated K562 clone 9.mbIL-21 at a ratio of two K562 clone 9.mbIL-21 cells per NK cell in medium containing 100 IU/mL recombinant human IL-2 (Biological Resources Branch, NCI, Frederick, MD, USA). NK cells were counted and re-suspended in fresh IL-2 supplemented medium every two–three days. Every seven days, NK cells were collected, counted, and plated with freshly irradiated feeder cells at a ratio of one K562 clone 9.mbIL-21 per NK cell. NK cells were grown in RF10 complete medium in upright T-75 flasks each containing no more than 40 mL total volume. All NK cells were used between days seven and 21 of expansion.

### 4.4. Bortezomib Treatment Assays

For bortezomib assays cells were plated in 10 mL of standard culture medium at cell densities of 1 × 10^6^ for U87 and SW982; or 0.5 × 10^6^ for PANC-1 into T-25 culture flasks. Bortezomib (Selleckchem, Houston, TX, USA) was then added to cell culture medium at the indicated concentrations (0 nM refers to untreated controls). At indicated time points, all live and dead cells were harvested by incubating cells with TrypLE (Thermofisher, Waltham, MA, USA) for 5 min at 37 °C, followed by neutralization and washing with fresh medium and used for further analysis or experimental assays. Cell viability was assessed by trypan blue exclusion using the TC20 automated cell counter (Bio-Rad, Hercules, CA, USA).

### 4.5. Flow Cytometric Cytotoxicity Assay

1 × 10^5^ tumor cells were plated in triplicate with the indicated ratios of NK cells in a 96-well plate for 24 h. After 24 h, plates were washed and analyzed using the ALDEFLUOR™ assay, as previously described [17,49]. All flow cytometric killing assays were additionally stained with CD45 to exclude NK cells from analysis and Fixable Viability Dye™ (eBioscience, San Diego, CA, USA) to exclude dead cells.

### 4.6. Flow Cytometry and Cell Sorting

Brilliant Violet 711 anti-CD45 (HI30), PE-CY7 anti-NKG2D (1D11) PerCPef710 anti-HLA-ABC (W6/32), APC anti-MICA/B (6D4), PECy7 anti-Fas (DX2), Brilliant Violet 605 anti-CD56 (HCD56), and PE DR5 (DJR2-4) were purchased from BioLegend (San Diego, CA, USA). Fixable Viability Dye 455UV and ef780 were purchased from eBioscience. Stained samples were acquired on an LSR Fortessa with High-throughput system attachment (HTS) (BD Biosciences, San Jose, CA, USA) and analyzed with FlowJo version 10 software (TreeStar, Ashland, OR, USA). ALDEFLUOR™ expression (STEMCELL Technologies, Vancouver, BC, Canada) was determined according to manufacturer’s instructions and with a DEAB (Diethyl Amino Benzaldehyde) control well for each Effector: Target ratio and for each individual condition tested accordingly. Cells were kept at 4 °C until analysis and were analyzed in small batches to prevent leakage due to changes in temperature. For fluorescence-activated cell sorting (FACS) sorting, cells were stained with ALDEFLUOR similar to flow cytometry analysis, and tubes were submerged on ice during and after sorting. About 2% of the brightest ALDEFLUOR cells and 5% of the dimmest ALDEFLUOR cells were gated and sorted into 500 μL of FBS using a Becton Dickinson Influx cell sorter (BD Biosciences, San Jose, CA, USA). After sorting, cells were washed with fresh medium and transferred to T-25 culture flasks and cultured under standard cell culture conditions until cells reached 80–90% confluency. Thereafter, cells were harvested, counted, and cryopreserved for future experiments.

### 4.7. Tumor Models

NSG (NOD.Cg-Prkdcscid Il2rgtm1Wjl/SzJ) mice were given tumors through subcutaneous or intracranial injections. For subcutaneous models, mice received 10^6^ U87 cells in 100 μL PBS in the rear flank. For the intracranial model, anesthetized NSG mice were injected with 5 × 10^4^ luciferase-transfected U87 cells orthotopically 2.5 mm left and 1 mm anterior to the bregma. A 10 μL Hamilton syringe was plunged 3.5 mm deep, then withdrawn 1 mm (to 2.5 mm) using a Just for Mice™ stereotaxic instrument (Braintree Scientific, Inc., Braintree, MA, USA) and 2 μL of total volume was injected. All experimental protocols were approved by the UC Davis Institutional Animal Care and Use Committee (Protocol #19621).

### 4.8. Hydrodynamic IL-15 Plasmid Delivery

For in vivo studies, we injected an IL-15-producing plasmid, which we have used previously to sustain the viability of NK cells in an immunodeficient host [50]. Expression of IL-15 in this plasmid is driven via the cytomegalovirus (CMV) promoter and human enhancer filamentation 1 (HEF-1) enhancer. Mice are injected via high pressure tail vein injection of 1.6 mL of PBS containing 10 μg of plasmid in 4–6 seconds. This hydrodynamic IL-15 gene delivery was always administered 24 h before NK cell injections.

### 4.9. In Vivo Imaging

Following implantation, tumor burden was assessed every three-four days. Animals were anesthetized with inhaled isoflurane and injected with 3 mg cold D-luciferin. After 5 min anesthetized mice were placed into an IVIS-Spectrum imaging station (Caliper Life Sciences, Waltham, MA, USA) and bioluminescence was recorded. Alternatively, mice were imaged by T2-weighted Magnetic resonance imaging (MRI) on a Biospec 7T (Bruker, Billerica, MA, USA) in collaboration with the UC Davis Center for Molecular and Genomic Imaging.

### 4.10. RNA Extraction and qPCR

Total RNA was extracted using the RNeasy plus mini kit (Qiagen, Venlo, Netherlands). RNA concentrations were then quantified using a Qubit4 Fluorometer (Invitrogen, Carlsbad, CA, USA) and RNA integrity was assessed using the Agilent TapeStation (Agilent, Santa Clara, CA, USA) Total RNA was reverse transcribed using iScript, and qPCR was performed using CFX384 (Bio-Rad, Hercules, CA, USA). Reference genes used for normalization were GAPDH and HPRT1. Data were analyzed using the Bio-Rad CFX manager software (Bio-Rad) and expressed as fold change. PrimePCR array (Bio-Rad) was performed following manufacturer’s instructions and recommendation using a CFX96 from the same vendor and data analysis performed using CFX Maestro (Bio-Rad).

### 4.11. Statistics

Statistical analysis was performed using Prism software (GraphPad Software Inc., San Diego, CA, USA). Data were expressed as mean ± (SEM) unless otherwise stated. For analysis of three or more groups, analysis of variance (ANOVA) tests were performed with a Bonferroni or Tukey post-hoc test, when appropriate. Analysis of differences between two normally distributed test groups was performed using the Student’s *t*-test. If *p* < 0.05, *p*-values were considered statistically significant.

## 5. Conclusions

These studies provide insight into the sensitization effect on CSCs caused by the proteasome inhibitor bortezomib, as this treatment yields an enrichment of an ALDH^bright^ subpopulation in different human cell lines, including PANC-1, SW982, and U87, as well as primary human breast and brain tumor cells. Moreover, bortezomib treatment results in increasing expression of the tumor necrosis factor (TNF) family receptors DR5 and Fas as well as the stress induced ligands MICA/B, which are targeted by the NKG2D receptor expressed by human activated NK cells. Taken together, these studies provide important information in favor of the use of NK cell adoptive transfer as an adjuvant to existing chemotherapeutic approaches utilizing bortezomib in chemorefractory tumors such as glioblastoma and sarcomas.

Further work is required to better elucidate optimal dosing and timing of bortezomib when used in combination with NK cell adoptive transfer. The optimal techniques for expanding and activating human NK cells remains an area of great interest given the potential therapeutic benefits for treatment of solid malignancies and successful elimination of CSC subpopulations.

## Supplementary Materials

The following are available online at http://www.mdpi.com/2072-6694/11/1/85/s1, Figure S1: Cell counts and number of ALDH^brigh^ and ALDH^dim^ from bortezomib treated cells, Figure S2: HLA-ABC expression in ALDH^bright^ and ALDH^dim^ cells treated with bortezomib, Figure S3: Bortezomib increases expression of cancer stem cells related genes and stress ligands on sorted ALDH^bright^ and ALDH^dim^ subpopulations in U87, Figure S4: Bortezomib increases ALDH^bright^ cells by frequency and numbers and decreases the frequency of viable cells in dog sarcoma.

## Abbreviations

ALDH	Aldehyde dehydrogenase
CSC	Cancer stem cell
NK	Natural killer
DR5	Death receptor 5
MIC A/B	MHC class I polypeptide-related sequence A/B
