# Supplementary material for: Bortezomib Augments Natural Killer Cell Targeting of Stem-Like Tumor Cells
Source: Cancers (Basel). 2019 Jan 14;11(1):85. doi: 10.3390/cancers11010085 (PMC6356940; doi:10.3390/cancers11010085)
Supplement: Supplementary file 1 [file cancers-11-00085-s001.pdf]

Supplementary Material

Bortezomib Augments Natural Killer Cell Targeting of Stem-Like Tumor Cells

Jesus I. Luna, Steven K. Grossenbacher, Ian R. Sturgill, Erik Ames, Sean J. Judge, Lyes A. Bouzid, Morgan A. Darrow, William J. Murphy and Robert J. Canter

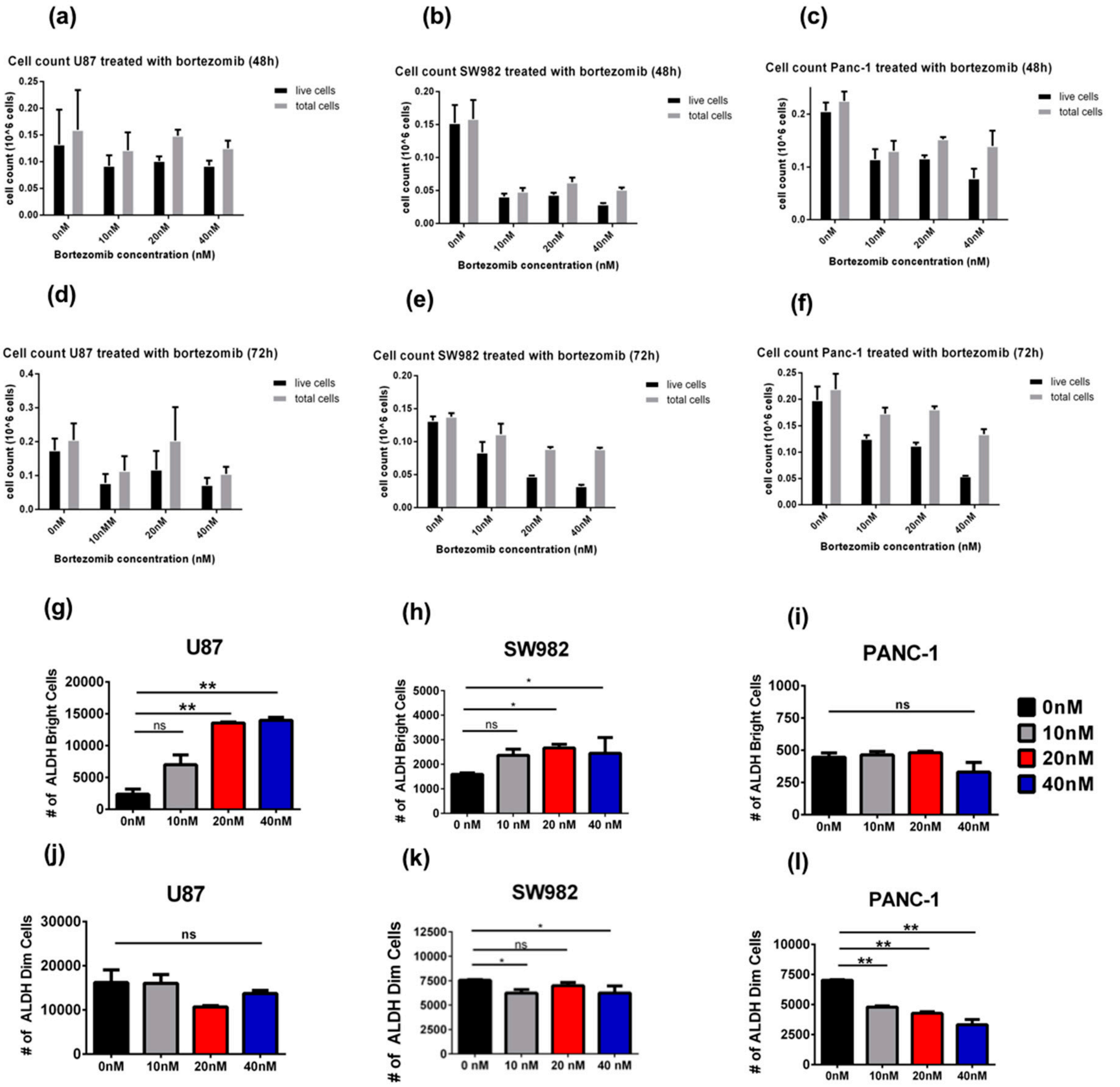

Figure S1. Cell counts and number of $\text{ALDH}^{\text{brigh}}$ and ALDH^{dim} from bortezomib treated cells. Cell counts indicating total cell numbers and total live cells in U87 (**a**,**d**), SW982 (**b**,**e**), and PANC-1 (**c**,**f**) after 48 and 72 h, respectively. Numbers of $\text{ALDH}^{\text{bright}}$ and ALDH^{dim} cells in U87 (**g**,**j**), SW982 (**h**,**k**), and PANC-1 (**i**,**l**) cells treated with 0, 10, 20, and 40 nM bortezomib for 48 h. Cell numbers were calculated by flow cytometry based on aldefluor assay. (* = $p < 0.05$, ** = $p < 0.01$).

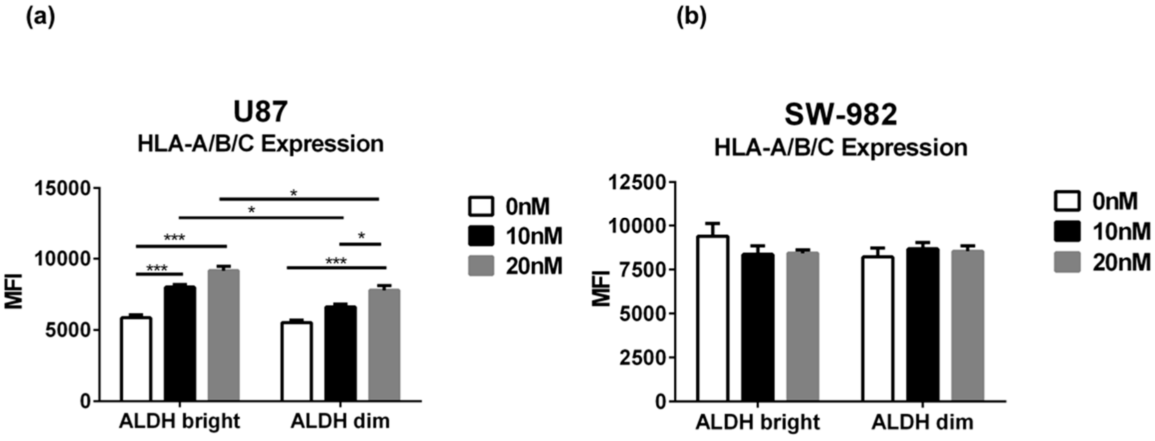

Figure S2. HLA-ABC expression in ALDH^{bright} and ALDH^{dim} cells treated with bortezomib. Bar graphs showing expression levels of HLA-ABC in U87 (**a**) and SW982 (**b**) treated with 0, 10, and 20 nM bortezomib for 48 h. (* = $p < 0.05$, ** = $p < 0.01$, *** = $p < 0.001$).

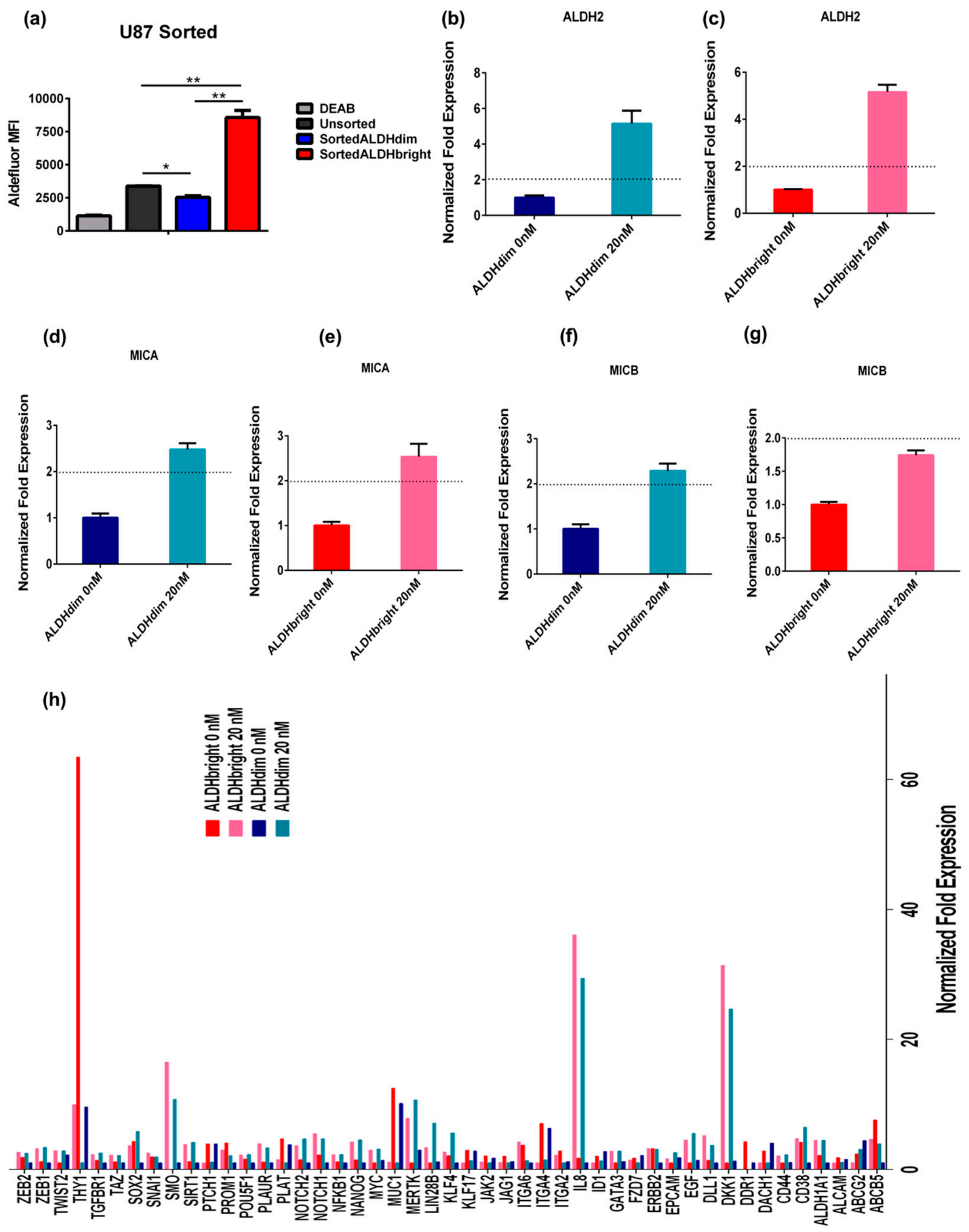

Figure S3. Bortezomib increases expression of cancer stem cell related genes and stress ligands on sorted ALDH^{bright} and ALDH^{dim} subpopulations in U87. (**a**) Bar graph showing MFI values comparing sorted ALDH^{bright} and ALDH^{dim} subpopulations, and unsorted cells including MFI for DEAB control. (**b**) Expression of *ALDH2* in ALDH^{dim} treated with 0 and 20 nM; and (**c**) ALDH^{bright} treated with 0 and 20 nM. (**d**,**f**) Expression of *MICA* and *MICB* in ALDH^{dim} treated with 0 and 20 nM; and (**e**,**d**,**g**) ALDH^{bright} cells treated with 0 and 20 nM. (**h**) Bar graph showing expression levels of cancer stem cell related genes in ALDH^{dim} treated with 0 and 20 nM and ALDH^{bright} treated with 0 and 20 nM. Dotted lines indicate a two-fold expression threshold as indication of significant gene up-regulation. (* = p < 0.05, ** = p < 0.01).

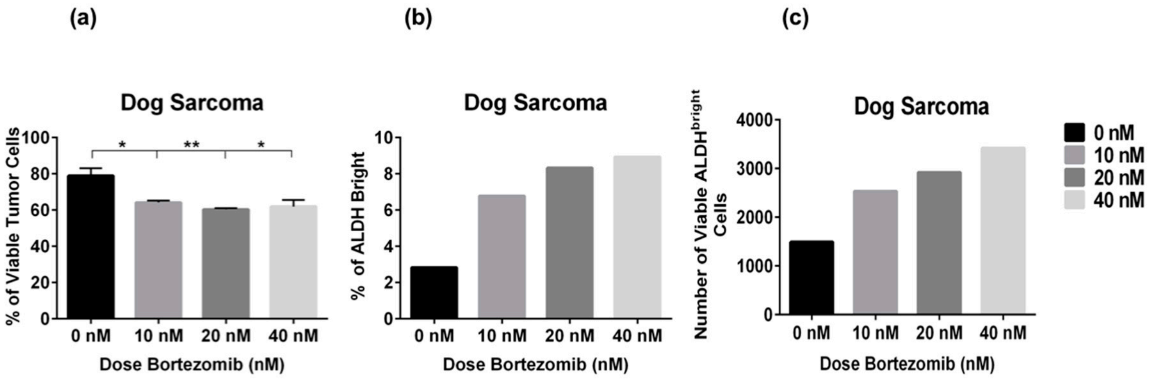

Figure S4. Bortezomib increases ALDH^{bright} cells by frequency and numbers and decreases the frequency of viable cells in dog sarcoma. (**a**) % of viable tumor cells, (**b**) % of ALDH^{bright} cells, and (**c**) number of ALDH^{bright} cells in dog sarcoma treated with bortezomib for 24 h. (* = $p < 0.05$, ** = $p < 0.01$).
